# The Nrf2/Keap1/ARE Pathway and Oxidative Stress as a Therapeutic Target in Type II Diabetes Mellitus

**DOI:** 10.1155/2017/4826724

**Published:** 2017-08-20

**Authors:** Joshua A. David, William J. Rifkin, Piul S. Rabbani, Daniel J. Ceradini

**Affiliations:** Hansjörg Wyss Department of Plastic and Reconstructive Surgery, New York University School of Medicine, 430 East 29th Street, New York, NY 10016, USA

## Abstract

Despite improvements in awareness and treatment of type II diabetes mellitus (TIIDM), this disease remains a major source of morbidity and mortality worldwide, and prevalence continues to rise. Oxidative damage caused by free radicals has long been known to contribute to the pathogenesis and progression of TIIDM and its complications. Only recently, however, has the role of the Nrf2/Keap1/ARE master antioxidant pathway in diabetic dysfunction begun to be elucidated. There is accumulating evidence that this pathway is implicated in diabetic damage to the pancreas, heart, and skin, among other cell types and tissues. Animal studies and clinical trials have shown promising results suggesting that activation of this pathway can delay or reverse some of these impairments in TIIDM. In this review, we outline the role of oxidative damage and the Nrf2/Keap1/ARE pathway in TIIDM, focusing on current and future efforts to utilize this relationship as a therapeutic target for prevention, prognosis, and treatment of TIID.

## 1. Introduction

The worldwide prevalence of diabetes mellitus (DM) was estimated at 8.5% in 2014, and the morbidity resulting from the microvascular and macrovascular complications of this disease is enormous [1]. Costs attributable to DM in 2013 were $101.4 billion in the United States alone, making it the most expensive medical condition by a significant margin [2–4]. The chronic hyperglycemia and impairments in insulin secretion and action that characterize type II diabetes mellitus (TIIDM) are associated with long-term damage, dysfunction, and failure of many organs, including the eyes, kidneys, nerves, heart, and blood vessels [5]. Decades of scientific research, randomized human trials, and clinical experience have demonstrated that a combination of lifestyle modifications and pharmaceutical interventions has the capacity to prevent or delay the onset of TIIDM and many of its devastating complications [6]. Consequently, major therapeutic advances, coupled with increased diabetes awareness and the implementation of national programs and guidelines for diabetes prevention, have helped alleviate disease-related morbidity and mortality [7]. Despite these improvements, this disease continues to pose a tremendous burden in the US, and the prevalence, deaths, and costs attributable to TIIDM are expected to continue increasing drastically [8]. As we gain further insight into the molecular underpinnings of this disease and its destructive sequelae, we advance the opportunity to develop novel, targeted approaches for diabetes treatment, prognosis, and, ultimately, prevention. In recent years, the Nrf2/Keap1/ARE antioxidant pathway has emerged as one such promising avenue of research. In this review, we summarize the roles of oxidative stress and the Nrf2/Keap1/ARE pathway in TIIDM, as well as the current state of efforts aimed at exploiting this relationship in order to minimize the devastating impact of this disease across the globe.

## 2. Oxidative Stress and Diabetes Mellitus

Oxidative stress occurs when free radical production overwhelms the endogenous antioxidant ability to neutralize these highly reactive chemical compounds. The ensuing cellular damage, such as DNA cross-linking and apoptosis, is a hallmark of oxidative stress and is a fundamental pathological process in cancer, aging, and a variety of chronic diseases [9–12]. In TIIDM, dysfunctional redox homeostasis has long been known to play a role in the pathogenesis of the disease and its complications through a variety of mechanisms, and diabetic patients have been shown to possess increased cellular levels of reactive oxygen species (ROS) and ROS-induced DNA damage [13–15]. Landmark studies by Giacco and Brownlee showed that the increased glycemic load in diabetes overwhelms the Krebs cycle, resulting in the inhibition of election transfer within the mitochondrial membrane and the accumulation of free radicals [14]. In particular, these free radicals include the highly reactive superoxide and hydroxyl compounds [16]. As ROS production increases, upregulation of four biochemical pathways occurs: polyol flux, intracellular advanced glycosylation end product (AGE) formation, protein kinase C activation, and hexosamine pathway flux [14]. These perturbations result in a variety of downstream effects known to underlie the pathogenesis and progression of TIIDM, including the depletion of natural antioxidant molecules and damage to vascular cells, as well as alterations in gene and protein expression, blood flow, and endothelial cell permeability [13, 17, 18].

## 3. The Nrf2/Keap1/ARE Pathway

**Figure 1:**
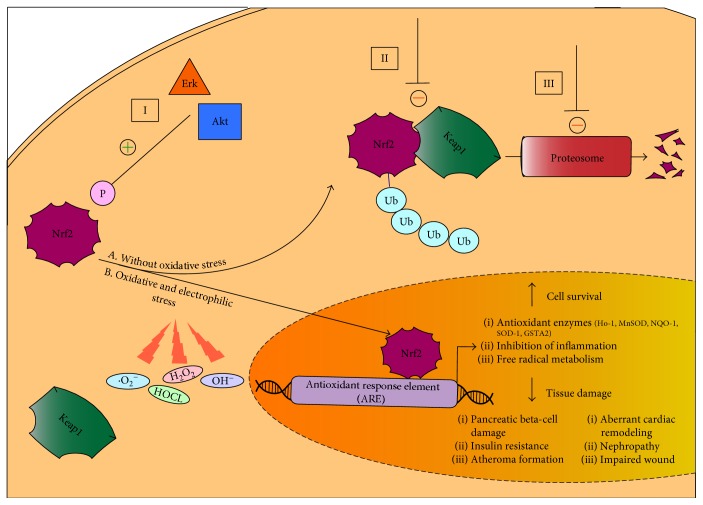
The Nrf2/Keap1/ARE pathway in type II diabetes mellitus. (A) Under nonstressed conditions, the Nrf2 transcription factor is covalently bound to cysteine residues on its native repressor Keap1 in the cytoplasm. This results in the constitutive ubiquitination and degradation of Nrf2 in the proteasome and inhibition of the anti-oxidant response. (B) Under conditions of electrophilic or oxidative stress, cysteine residues on Keap1 are modified, resulting in the stabilization and translocation of Nrf2 into the nucleus, where it can bind to the promoter region of the ARE and initiate the transcription of various cytoprotective enzymes which function to promote cellular survival through a variety of mechanisms, including the upregulation of antioxidant function, inflammatory inhibition, and the transport of toxic metabolites. These cellular adaptations have been shown to improve a wide array of tissue damage underlying the pathogenesis and progression of diabetes. (B) There are three major mechanisms of Nrf2 induction by small molecule activators. (I) Upstream kinases such as Akt and Erk phosphorylate Nrf2 at specific sites, favoring its release by Keap1 and nuclear translocation. (II) Modification of Keap1 cysteine residues disrupts the Nrf2-Keap1 complex, favoring dissociation of Nrf2 and subsequent nuclear translocation. (III) Inhibition of Nrf2 ubiquitination by Keap1 and degradation by the proteasome augments Nrf2 availability, thus favoring nuclear translocation of Nrf2. Ub: ubiquitination; P: phosphorylation.

Knowledge of the relationship between oxidative stress and TIIDM has precipitated intense investigation into the failure of the diabetic system to appropriately respond to increased oxidative loads. Regulation of cellular redox homeostasis under both stressed and nonstressed conditions occurs primarily at the transcriptional level, and the Nrf2/Keap1/ARE pathway is the primary mediator of this response. This signaling pathway regulates the expression of over 100 genes and functions related to oxidative stress and cell survival, including direct antioxidant proteins, phase I and II electrophile detoxification enzymes, the transport of toxic solutes, free radical metabolism, inhibition of inflammation, glutathione homeostasis, proteasome function, and the recognition of DNA damage, as well as the expression of various related growth factors and transcription factors [19] (Figure 1).

The principal mediator of this response is nuclear factor E2-related factor 2 (Nrf2), a master transcription factor. Upon binding the upstream cis-regulatory antioxidant response element (ARE) sequence located in the promoter regions of cytoprotective genes, Nrf2 triggers the transcriptional induction of multiple detoxifying enzymes [20]. Under nonstressed conditions, Nrf2 activity is suppressed by its native repressor Kelch-like ECH-associated protein 1 (Keap1), through interactions with a hairpin motif in the C terminus of the Nrf2-ECH homologous domain (Neh2) phosphorylation site on Nrf2. Keap1 is a cytoplasmic, actin cytoskeleton-associated adapter protein of the Cullin3- (Cul3-) based E3-ligase complex, which tags Nrf2 for ubiquitination and subsequent proteosomal degradation within the cytoplasm [21]. This signaling pathway has been established as the major mechanism of cellular defense against oxidative stress both physiologically and in a wide array of disease models [19]. First isolated in cloning studies in 1994 [22], the critical role of Nrf2, a member of the cap'n'collar family of basic leucine zipper transcription factors, in both constitutive and inducible ARE gene expression was soon elucidated both *in vitro* [23] and *in vivo* [20, 21, 24]. The precise molecular mechanisms of Nrf2 and Keap1 interaction are a topic of debate, particularly given the distinct subcellular locations of these two molecules [25]. It is nonetheless understood that modification of cysteine residues in the primary structure of Keap1, which act as cellular sensors for inducers of environmental stress, by thiol-reactive chemical species during states of excess oxidative or electrophilic stress results in the disruption of the Nrf2-Keap1 dimer and stabilization of Nrf2 [26]. Nrf2, once stabilized, is no longer repressed by Keap1 and becomes free to heterodimerize with members of the Maf family of transcription factors. With the assistance of a nuclear localization sequence, the Nrf2 heterodimer can rapidly translocate into the nucleus and bind to the ARE, resulting in the recruitment of elements required for the transcriptional activation of a variety of genes such as glutathione S-transferase A2 (GSTA2), NADPH quinone oxidoreductase (NQO-1), superoxide dismutase (SOD1), and heme oxygenase-1 (Ho-1) [19, 20, 27]. These antioxidant enzymes function to transform free radicals into less toxic substances through four primary mechanisms: (1) oxidation and reduction reactions, in which functional groups on hydrophobic molecules are exposed, (2) nucleophilic trapping processes, (3) transporter efflux of toxic metabolites, and (4) maintenance of reduced conditions by thiol-containing molecules [19, 28]. This protective stress recognition mechanism by Keap1 dually ensures suppression of Nrf2 during nonstressed conditions and an appropriate antioxidant response during periods of excessive cellular stress.

**Figure 2:**
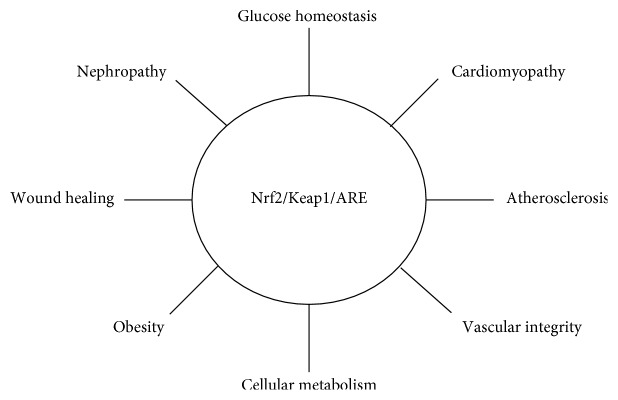
The Nrf2/Keap1/ARE pathway is involved in multiple tissue types.

Given the intimate relationship between TIIDM and oxidative damage, the involvement of the Nrf2/Keap1/ARE pathway in this unsolved clinical problem has become a topic of great interest. We now know that dysfunction of this master antioxidant pathway is associated with the pathophysiology of diabetes and a wide range of its complications, such as diabetic nephropathy and impaired cutaneous wound healing, in both animal and human models [29–31] (Figure 2).

While the mechanism or mechanisms of this dysfunction in diabetes have only begun to be elucidated, therapies targeting the Nrf2/Keap1/ARE pathway represent a promising avenue in current research. As a critical upstream mediator of not only the global antioxidant response but also of anti-inflammatory genes and transcription factors involved in mitochondrial function, the Nrf2/Keap1/ARE pathway represents an ideal target in treating the widespread oxidative damage implicated in pancreatic damage, insulin resistance and sensitivity, and the progression of a broad spectrum of diabetic complications. Additionally, the inducible nature of this signaling pathway allows Keap1 to uniquely both sense the cellular redox state and responsively modify the degree of Nrf2 degradation via ubiquitination in response to this oxidative stress. This allows for modulation of cellular redox homeostasis via highly specific transcriptional activation of only those genes containing an ARE in the promoter region [32]. Furthermore, the Nrf2-mediated oxidative response may also possess aspects that are specific to diabetes. The activation of the aforementioned pathways underlying the pathogenesis of diabetic complications has been tied to a singular hyperglycemia-induced event in the mitochondria, overproduction of superoxide by the electron-transport chain [13]. Furthermore, studies have shown that the absence of Nrf2 may exacerbate both type I and type II diabetes [33, 34]. This hyperglycemia-specific increase in ROS overproduction by the mitochondria may explain why classic antioxidants, low molecular-weight compounds that can scavenge reactive oxygen intermediates, have not been proven beneficial in the treatment of diabetic complications. In contrast to these classic or direct antioxidants, the battery of cytoprotective agents that are upregulated by the Nrf2/Keap1/ARE pathway have been termed “ultimate antioxidants,” which possess long half-lives, are not depleted throughout the course of their wide range of chemical detoxification reactions, and can even accelerate regeneration of other antioxidants, such as glutathione [35]. Lastly, the ability to target this pathway at a variety of locations, as will be discussed later, grants an incredibly rich degree of flexibility and diversity as the search for rational and clinically relevant therapeutics evolves.

## 4. Nrf2/Keap1/ARE and the *β*-Cell

Pancreatic *β*-cell dysfunction and the resulting impairments in insulin sensitivity and production are a critical component in the development and progression of both type I and type II DM [36], and oxidative stress is one important mechanism whereby this damage occurs [37]. Despite overexpression of Nrf2 downstream endogenous antioxidant genes by the pancreas in order to curtail cellular damage and salvage insulin-secreting ability, oxidative damage ultimately overloads this protective response in TIIDM [38–40]. In humans, these deleterious effects manifest as reduced *β*-cell mass and DNA damage in the pancreatic islets of patients with TIIDM [41]. Reversing the decline and eventual failure of pancreatic *β*-cells is critical for preventing TIIDM and its progression [42].

Animal studies have shown that the Nrf2/Keap1/ARE system is a crucial defensive pathway in the physiological and pathological protection of pancreatic *β*-cells. In *β*-cell-specific transgenic mice, Nrf2 depletion depressed the expression of cytoprotective antioxidant genes in pancreatic islets and exacerbated oxidative *β*-cell damage, while Nrf2 induction suppressed the accumulation of intracellular ROS, the formation of ROS-induced DNA adducts, and pancreatic *β*-cell apoptosis within the islets [43]. Further studies showed the preservation of *β*-cell mass and function in diabetic mice with genetically modified upregulation of Nrf2 via Keap1 knockout [44]. Pancreatic *β*-cell protection by the Nrf2/Keap1/ARE system is not limited to free radical scavenge but includes reduction of inflammation via the NF-kappaB pathway [45] and maintenance of critical cellular degradation systems such as apoptosis, autophagy, and proteosomal degradation [46, 47].

In addition to *β*-cell injury, oxidative stress also affects pancreatic insulin secretion, although this relationship is less clear. While some studies show that ROS impairs insulin release through mechanisms such as a reduction in ATP production [48] and increased glyceraldehyde 3-phosphate dehydrogenase (GAPDH) activity [49], there is a growing body of evidence indicating that oxidative and electrophilic stress can actually augment, and may even be necessary for, insulin release [50, 51]. The role of the Nrf2/Keap1/ARE pathway in insulin secretion is similarly controversial. While insulin content and secretion are decreased in the pancreatic islets of Nrf2 knockout mice and upregulation of Nrf2 appears to improve the insulin-releasing potential of *β*-cells [44, 52, 53], Nrf2 deficiency has also been associated with decreased blood glucose, enhanced insulin signaling, and decreased fat and body weight in Nrf2 knockout mouse models [54–57]. Clearly, a great deal of work remains before we completely understand the role of oxidative stress and Nrf2 with regard to glucose and insulin homeostasis. Of note, while most studies have focused on the role of Nrf2/Keap1/ARE on glucose and insulin handling within *β*-cells, emerging evidence suggests that this pathway may also play a dynamic role in other pancreatic islet cells (*α*-cells, *δ*-cells, and PP-cells), possibly by preventing differentiation of *β*-cells into these insulin-negative cell types under conditions of oxidative stress [58].

## 5. Nrf2 and Insulin Resistance

In addition to *β*-cell dysfunction, insulin resistance in a wide range of tissues is a hallmark of TIIDM, resulting in elevated blood glucose levels and exacerbation of pancreatic damage as it attempts to compensate for perceived hypoglycemia. Studies by Uruno et al. in murine models of Nrf2 over-expression via both genetic Keap1 knockdown and pharmacological induction suggest that Nrf2 activation can improve insulin sensitivity in diabetes and abrogate diabetes and obesity in mice [44]. Body weight and blood glucose levels were decreased in diabetic mice with Keap1 knockout. These findings were attributed to Nrf2-mediated stimulation of energy consumption in skeletal muscle and brown adipose tissue. Further studies showed that Nrf2 induction in mice also suppressed gluconeogenesis, owing to transcriptional repression of a variety of enzymes including the gluconeogenic enzyme glucose-6-phosphatase (G-6-P). *In vitro* studies using murine hepatocytes confirmed that Nrf2 attenuates G-6-P expression in these cells, despite stimulation of gluconeogenesis using a cAMP analog. In addition to its inhibitory effect on G-6-P, Nrf2 was shown to decrease expression of other genes related to gluconeogenesis, as well as augment insulin sensitivity in an insulin tolerance test. A more recent study in a murine model suggests that increased Nrf2 signaling may also improve insulin resistance via suppression of oxidative stress in the hypothalamus, a phenomenon that may affect systemic metabolic regulation [59]. Furthermore, obesity is associated with an increased risk of developing insulin resistance and TIIDM, and murine studies have likewise shown that Nrf2 induction can suppress weight gain and increase skeletal muscle oxygen consumption, mitochondrial redox homeostasis, and ATP production, as well as augment cellular glucose uptake [44, 60, 61].

## 6. Diabetic Complications (Table 1)

### 6.1. Cardiovascular Disease

The absolute risk of cardiovascular disease (CVD) is 2-fold greater in patients with TIIDM versus those without, and at least 65% of people with DM die of heart disease or stroke [3, 62]. Given this close link between TIIDM and CVD, it is not surprising that oxidative stress has been implicated in the pathogenesis of many CVD disorders, including hypertension [63], heart failure (HF) [64], atherosclerosis [65], and ischemia-reperfusion injury [66]. Free radical-induced endothelial damage is thought to be the initiating step in CVD [67], and hyperglycemia-induced ROS in TIIDM exacerbates impairments in angiogenesis and neovascularization through means such as disruption of endothelial progenitor cell function and vascular homeostasis [68, 69]. The importance of Nrf2 and its downstream elements to vascular integrity has also become increasingly apparent, and studies have illuminated their role in functions such as augmentation of blood vessel branching [70], preservation of endothelial cell function [71], blood pressure regulation [72], and protection of the myocardium following ischemia [73, 74]. In particular, evidence of Ho-1, the Nrf2 downstream enzyme that catalyzes the degradation of heme into biliverdin, ferrous iron, and carbon monoxide, as an important mediator against vascular dysfunction in diabetes has recently emerged. Upregulation of Ho-1 levels was shown to improve left ventricular ejection fraction and inhibit remodeling in diabetic rats with myocardial infarction, and *in vivo* and *in vitro* studies demonstrated that Ho-1 overexpression attenuated angiotensin II-mediated cardiac hypertrophy in these mice [74]. Studies in streptozotocin-induced diabetic mice suggest that these effects are due to reductions in oxidative stress, inflammation, and apoptosis [75]. Therefore, the likely link among ROS and the Nrf2/Keap1/ARE pathway to the plethora of CVD-related complications that afflict diabetic patients has emerged as a subject of intense investigation.

### 6.2. Atherosclerosis

Nrf2 has been implicated in a variety of processes intrinsic to the formation of atherosclerotic plaque. As an indispensable component of the antioxidant response within macrophages [76], Nrf2 protects these phagocytic cells from oxidized low-density lipoproteins (oxLDL) and foam cell transformation, fundamental steps in atheroma formation [77, 78]. Additionally, Nrf2 appears to inhibit the proinflammatory response in endothelial cells located at atherosusceptible sites, conferring them a protective advantage in response to diabetic hyperglycemia [71, 79]. The significance of these effects is largely attributed to the downstream activation of the Nrf2 downstream antioxidant enzyme HO-1, which has been independently found to play a significant defensive role against atherosclerosis [80]. However, there are also studies suggesting that Nrf2 can promote atheroma formation, possibly due to interactions with a variety of well-described proatherogenic factors such as vascular cell adhesion molecule 1 (VCAM-1) and interleukin-1 (IL-1) [81–83]. For instance, overexpression of Ho-1 was found to be associated with worsening coronary atherosclerosis in an autopsy study of Japanese patients with diabetes mellitus [84]. This seeming contradiction might be explained by a differential response of Nrf2 to laminar versus oscillatory blood flow, as atheroma formation is not uniform throughout the vascular system but rather disposed to bifurcation and branch points [85]. Regardless, mounting evidence of the importance of Nrf2 for vascular integrity and long-term endothelial function suggests that the Nrf2/Keap1/ARE pathway is influential in atherosclerotic resistance and may be a useful target for protection against coronary artery disease (CAD), peripheral vascular disease, and cerebrovascular disease in diabetic populations.

### 6.3. Heart Failure

Not only is there a well-established association between TIIDM and the development of HF, but this relationship also persists even in the absence of other risk factors such as CAD or hypertension, suggesting that TIIDM may mediate an exclusive form of cardiomyopathy [86]. Multiple mechanisms, such as impaired regulation of intracellular calcium and accumulation of AGE products, have been suggested to underlie this dysfunction, all of which ultimately result in oxidative stress and myocardial toxicity [87]. Not surprisingly, ROS and mitochondrial dysfunction are increased in the diabetic heart [64]. The resulting cardiac cellular necrosis and apoptosis impair contractile and electrical function, two contributing features of HF [88–90]. In addition to causing cellular damage, ROS can modify proteins essential in excitation-contraction coupling [91], activate hypertrophy-signaling kinases [92], and stimulate cardiac extracellular matrix (ECM) remodeling [93]. Furthermore, ROS exacerbates insulin resistance of myocytes, a key element of diabetes-induced cardiac dysfunction [94].

Evidence that aberrant cardiac remodeling is attenuated by a variety of Nrf2 target genes such as SOD [95], HO-1 [96] and glutathione peroxidase (GPx) [97] has motivated investigation into a possible protective role of the Nrf2/Keap1/ARE pathway against cardiomyopathy in DM. In murine models of HF and TIIDM, oxidative stress attenuates the expression of Nrf2 in cardiomyocytes and downregulates glucose utilization, resulting in insulin resistance [98]. Furthermore, Nrf2 overexpression diminishes ROS and myocardial hypertrophy, an effect that was facilitated by extracellular signal-related kinase (Erk), which normally acts to activate Nrf2 during oxidative stress [99]. It has therefore may be that Erk-mediated Nrf2 downregulation may underlie individual susceptibilities to CVD-related diabetic complications.

A number of studies utilizing pharmacologic Nrf2 activators have implicated the antioxidant properties of the Nrf2/Keap1/ARE pathway in cardioprotection [100, 101]. Consequently, there is a great deal of interest in therapeutically targeting this pathway as a means of preventing or reversing pathological cardiac remodeling [102] or delaying ventricular failure, the hemodynamic hallmark of HF in TIIDM [103, 104]. However, chronic overactivation of the Nrf2/Keap1/ARE signaling pathway may actually contribute to cardiomyopathy, which undermines the encouraging results of acute Nrf2 induction [105]. A long-term phase III clinical study in TIID patients with stage 4 chronic kidney disease (CKD) (BEACON trial) was terminated early due to a higher rate of cardiovascular events in the treatment group [106]. It is unclear as to why these adverse events were observed in this study and not in an earlier clinical trial in patients with stage 3 CKD (BEAM trial), but possibilities include significantly longer length of drug exposure or the use of a 20 mg fixed dose as opposed to an adjustable dosage [107]. However, whether this truly reflects a cardiomyopathic tendency of Nrf2 or is alternatively the result of other factors, such as an inherently increased rate of cardiovascular events in patients with more severe CKD, remains unknown.

### 6.4. Diabetic Nephropathy

Diabetic nephropathy is a well-known microvascular complication of chronic hyperglycemia, and both oxidative stress and an impaired response by the Nrf2/Keap1/ARE system have been implicated in its progression via renal cell apoptosis, fibrosis, and deficiencies in cellular regeneration [11, 108, 109]. In a streptozocin- (STZ-) induced mouse diabetes model, Nrf2 activation with sulforaphane suppressed nephropathy and significantly improved metabolic indices associated with TIIDM, such as hyperglycemia, polydipsia, polyuria, and weight loss [110]. These benefits can be largely attributed to decreases in renal oxidative and nitrosative stress, which act to reverse dysfunction in multiple known mediators of diabetic nephropathy such as transforming growth factor beta (TGF-B), ECM proteins such as fibronectin and collagen IV, and p21, a cell-cycle regulator [111]. Similar findings with other known activators of the Nrf2/Keap1/ARE pathway, such as resveratrol and MG-132, support the therapeutic targeting of this system to ameliorate the oxidative damage and glucose-induced mesangial cell proliferation, inflammation, and fibrosis which underlies diabetic nephropathy [112, 113]. In humans, decreased levels of Nrf2 and expression of target genes in the peripheral blood of patients with CKD further support the contribution of an impaired Nrf2 antioxidant signaling pathway to systemic oxidative overload and inflammation in diabetic nephropathy [114].

### 6.5. Wound Healing

Impaired wound healing is a well-known and devastating complication of TIIDM and represents the leading cause of chronic wounds and lower extremity amputations in the US [115]. However, despite adherence to tight control of blood glucose levels and advances in synthetic and biologic healing modalities, chronic wounds persist in diabetic patients, suggesting a more fundamental pathology in the diabetic regenerative milieu [116]. As in other diabetic complications, oxidative stress is important for the development of chronic wounds, and AGE in the diabetic wound microenvironment appear to impair wound contraction and remodeling, the inflammatory response, and ECM proliferation. [117] Several natural Nrf2 activators have shown a promise in treating diabetic wounds, and early induction of the Nrf2 pathway through the rhomboid family protein RHBDF2 accelerated cutaneous wound healing in mice [118, 119]. Interestingly, recent work in tissue regeneration models has demonstrated that hyperglycemia in diabetes is associated with Keap1 dysfunction, which prevents nuclear localization of Nrf2 and thus is an appropriate stress response [120]. Utilizing a cutaneous gene therapy model, these studies showed that small-interfering RNA (siRNA) targeted at Keap1 restored wound redox homeostasis, accelerated healing, and counteracted impairments in angiogenesis and reepithelialization, two critical functions of wound healing disrupted in diabetes, by restoring Nrf2 localization. This seems to support the notion that aberrant overexpression of Keap1 and resulting Nrf2 repression is a possible mechanism of the redox homeostasis dysfunction and impaired wound healing in diabetes. Whether this relationship extends to other aspects of the disease, and the relative contribution of Nrf2 to specific wound healing functions, remains to be seen.

## 7. The Nrf2/Keap1/ARE Pathway as a Therapeutic Target

Given the broad accountability of oxidative stress for many pathological processes, the Nrf2/Keap1/ARE system has emerged as a logical therapeutic target for the prevention or treatment of disease. This pathway has been studied most intensively in cancer [19] but also in chronic obstructive pulmonary disease (COPD) [121], neurodegenerative disorders [122], and autoimmune diseases such as inflammatory bowel disease (IBD) [123] and rheumatoid arthritis [124]. A multitude of clinical trials has also been pursued in order to assess the efficacy of targeting or modifying elements of the pathway in order to diminish ROS-induced damage in human disease [125–128]. As a critical upstream mediator of the pathway, Nrf2 induction has formed the basis of most of this research. There are three primary mechanisms by which current pharmacological activators increase Nrf2 expression (Figure 2). These consist of (1) activation of upstream kinases such as protein kinase B (Akt) and extracellular signal-regulated kinases (Erk), which phosphorylate specific sites favoring the release of Nrf2 from Keap1; (2) modification of Keap1 cysteine residues, which disrupts the Nrf2-Keap1 complex and favors Nrf2 dissociation; and (3) blockage of ubiquitination and/or proteosomal degradation of Nrf2 [129]. The end result of all of these mechanisms is Nrf2 stabilization and subsequent translocation into the nucleus, where it can exert its transcriptional effects and commence an antioxidant cascade. Of note, Nrf2 activators have already made their way into clinical practice; in 2013, dimethyl fumarate (BG-12, brand name Tecfidera®) was approved by the FDA for the treatment of multiple sclerosis and is thought to exert its therapeutic effects via augmentation of Nrf2's downstream cytoprotective, anti-inflammatory, and antioxidant properties [130, 131]. These achievements represent a promise that the Nrf2 pathway can be effectively used in other diseases in which oxidative stress plays a major role, such as TIIDM.

With regard to TIIDM, knowledge of the pivotal role of oxidative stress in the pathogenesis and progression of the disease originally precipitated investigation into natural antioxidants, such as vitamin E, vitamin C, and coenzyme Q10, as logical initial approaches [132–134]. However, results of these studies have generally been disappointing, and human clinical trials have not shown any benefit of organic molecules as adjunct therapies in preventing or treating diabetic complications [135–137]. Therefore, over the past decade, a significant amount of research, predominantly utilizing high throughput cell-based screening assays, has been devoted to identifying clinically applicable small molecule activators or inducers of endogenous antioxidant mechanisms, such as the Nrf2/Keap1/ARE pathway [138]. These “new mechanism-based antioxidants” have emerged as the new frontier of defense against oxidative stress and inflammation in TIIDM.

An array of Nrf2 small molecule activators, both natural and synthetic, has been identified and studied extensively (Table 2).

These include sulforaphane, curcumin, cinnamaldehyde, pterostilbene, oltipraz, and resveratrol. Some of the most encouraging candidates fall under the category of synthetic triterpenoids; triterpenoid, 2-cyano-3,12-dioxooleane-,1,9(11)-dien-28-oic acid (CDDO), and its derivatives have yielded highly promising results in animal models of heart failure [102], insulin resistance [52], and obesity [155]. Bardoxolone methyl (CDDO-Me), one such derivative originally developed as an anticancer drug, was incidentally found to exhibit renoprotective effects and has made its way into human trials. A phase II clinical trial (BEAM) in adults with TIIDM and advanced CKD showed significant improvements in glomerular filtration rate (GFR) with only mild side effects such as muscle spasms, hypomagnesemia, and gastrointestinal distress [107]. Unfortunately, a subsequent phase III trial (BEACON) in patients with TIIDM and stage IV CKD was terminated early due to serious adverse events [106].

In addition to systemic administration, targeted delivery systems represent a potential approach to treatment of localized diabetes complications. For example, an engineered lipid-protein system (lipoproteoplex) demonstrated safe and efficient delivery of si*Keap1* to diabetic wounds and resulted in accelerated wound healing [156]. Such novel delivery systems could also potentially circumvent the known toxicities resulting from covalent modifications (e.g., alkylation) of Keap1 cysteine residues that form the basis of function for many reported Nrf2 stabilizers [157]. Therefore, Nrf2 pathway modulation via direct, noncovalent inhibition of Nrf2-Keap1 protein-protein complexes is emphasized in current research. Additionally, strategies aimed at adjacent or downstream elements in the Nrf2 pathway have also gained traction as an alternative or combinatorial approaches to treatment. For instance, repression of BTB domain and CNC homolog (Bach1), a nuclear inhibitor of Nrf2, in combination with traditional Nrf2 activators, has shown promising results in neurodegenerative disease models for safely increasing the efficiency and biological activity of these agents [158].

In addition to the pathogenesis of TIIDM and its major complications, treatment of the metabolic alterations in TIIDM has also become a focus of intense investigation. Shin et al. found that Nrf2 regulates fibroblast growth factor 21 (FGF21), a key mediator of glucose and lipid metabolism, in mice [159]. This may have implications in guiding treatment of obesity in TIIDM, which is itself regulated, at least in part, by Nrf2 action on lipogenic gene expression and fatty acid synthesis [156]. Studies have shown that Nrf2/Keap1/ARE activators can exert potent reductions in body weight and hepatic fat accumulation in mice with an excellent safety profile and tolerance, representing potential novel, noninvasive options for managing obesogenesis in TIIDM [156, 160].

As we learn more about the protective aspects of the Nrf2 pathway, we must also appreciate the potential hazards of targeting Nrf2 as a therapeutic means. Accruing evidence points to a “dark side” of Nrf2, which also regulates cell proliferation [161]. Nrf2 and its downstream genes are overexpressed in many cancer cell lines and human cancer tissues, and ove-activation of this pathway appears to contribute to the evolution of cancer and chemoresistance of cancer cells [162, 163]. Furthermore, mutations in Nrf2 and Keap1 have been found in a large percentage of malignancies [164, 165]. These findings should not impede our pursuit of targeting the Nrf2/Keap1/ARE pathway in the treatment of diabetes but should rather encourage a cautious and vigilant approach.

## 8. Nrf2 as a Potential Biomarker

Although biomarkers as a means of noninvasive disease prediction or prevention have been a topic of intense investigation for many years, very few have made their way into the clinical setting, and utility is largely limited to ad hoc corroboration of pathologic events, such as myocardial ischemia and heart failure. An increasing number of studies have been published on biomarkers of oxidative stress in a wide array of human disease [166], and AGE [167], nitrotyrosine [168], preoxiredoxin [169], and 8-hydroxy-2′-deoxyguanosine (8-OhdG) [170] levels in the skin, plasma, and urine samples have been investigated for both type I and type II DM. However, lack of validation for these markers remains a major obstacle to clinical utility [171]. The adoption of Nrf2, or elements of the Nrf2/Keap1/ARE pathway, as potential biomarkers has been proposed for prognostic purposes in cancer and neurodegeneration [172, 173]. The pursuit of using the Nrf2 pathway as a biomarker for TIIDM and its complications remains in its infancy, but existing evidence suggests that there may indeed be utility in prediabetic and diabetic patients, and research is likely to accelerate in this field as the medical landscape continues to shift towards a front-end, preventative approach with regard to chronic disease management [31, 174].

## 9. Conclusion

We have answered many questions regarding the role of the Nrf2/Keap1/ARE pathway and oxidative stress in TIIDM, but more remain before we can capitalize on this relationship to attain widespread, clinical significance in humans. For instance, much of what we know is derived from animal studies, and it is unclear as to what extent the murine antioxidant system reflects that of humans. Additionally, despite clear evidence of dysfunction in the Nrf2/Keap1/ARE antioxidant response across a wide range of tissue types and disease stages in TIIDM, the specific mechanisms underlying Nrf2 dysfunction have yet to be fully elucidated. We must also be cognizant of alternative, potentially confounding effects that independent players within Nrf2/Keap1/ARE pathway may exert. For instance, in addition to its cytoprotective role against oxidative stress as part of the Nrf2/Keap1/ARE pathway, accumulating research indicates that the Nrf2 molecule can also independently control the expression of genes responsible for many aspects of cellular metabolism. These studies, largely dependent on transgenic diabetic and Nrf2 knockout murine models, have implicated this molecule in TIIDM pathogenesis, prevention, and progression, via means that are wholly distinct from its role in oxidative protection. For instance, Nrf2 regulates blood glucose homeostasis and metabolic reprogramming by redirecting anabolic pathways [175], inhibiting lipogenesis [176], and promoting insulin sensitization, thereby ameliorating insulin resistance [177]. A more robust understanding of these varied roles may help explain seeming paradoxes in the role of the Nrf2/Keap1/ARE pathway such as what we see in insulin homeostasis and atheroma formation. These examples highlight not only the pleotropic effects of Nrf2 throughout tissues but also the variety of ways in which it responds and interacts, often contradictorily, with different types of stress.

Looking towards the future, we must continue to validate the Nrf2/Keap1/ARE pathway as a mediator of the oxidative stress underlying TIIDM and further explore this role in less morbid complications such as retinopathy and neuropathy. As we continue to screen for and develop ways to target the Nrf2/Keap1/ARE pathway, the importance of identifying novel delivery systems and nontoxic mechanisms of Nrf2 activation will accelerate translation of these therapeutics into human trials. Finally, encouraging evidence for the use of the Nrf2/Keap1/ARE as a biomarker should catalyze efforts to validate its use in the clinical setting. The intertwining roles of Nrf2, oxidative stress, and TIIDM will continue to provoke interest for a time to come, but it is becoming increasingly clear that further understanding this intimate relationship has preventative, prognostic, and therapeutic value in combatting this devastating disease.

## Figures and Tables

**Table 1:** Nrf2/Keap1/ARE and diabetic complications.

Diabetic complication	Pathogenesis	Nrf2- (or downstream-) mediated effects
Atherosclerosis	(i) oxLDL formation	(i) Protection from oxLDL transformation of phagocytic cells [77, 78]
	(ii) Proinflammatory response in endothelial cells	(ii) Inhibition of proinflammatory response at atherosusceptible sites [71, 79]

Heart failure	(i) Aberrant cardiac and ECM remodeling	(i) Blood pressure regulation [72]
	(ii) Insulin resistance of myocytes	(ii) Protection of myocardium following ischemia
	(iii) Impaired regulation of intracellular calcium	(iii) Diminishes ROS and myocardial hypertrophy [99]
	(iv) Accumulation of AGE products	

Diabetic nephropathy	(i) Renal oxidative and nitrosative stress	(i) Improvement of metabolic indices (e.g., polydipsia and polyuria) [110]
	(ii) Mesangial cell proliferation, inflammation, fibrosis	(ii) Reversal in dysfunction of key growth factors and ECM proteins [111–113]

Wound healing	(i) Keap1 overexpression	(i) Impairments in angiogenesis and reepithelialization [120]
	(ii) Loss of wound redox homeostasis	
	(iii) Chronic inflammatory microenvironment	

**Table 2:** Small molecule activators of Nrf2 in TIIDM.

Molecule	Source	Mechanism of Nrf2 activation	Evidence
Sulforaphane (SFN)	Natural (cruciferous vegetables such as broccoli, brussel sprouts, and cabbage)	Modification of Keap1 cysteine residues	(i) Pancreatic *β*-cell protection [45]
			(ii) Prevented cardiac oxidative damage, inflammation, and hyperglycemic-induced fibrosis [139]
			(iii) Renal protection in db mice [110]

Curcumin (CUR)	Natural (turmeric)	Modification of Keap1 cysteine residues	(i) Reduced number of prediabetic individuals who progressed to type II DM [140]
			(ii) Activates liver enzymes involved in glycolysis, gluconeogenesis, and lipid metabolism [141]

Bardoxolone methyl (CDDO-Me/RTA 402)	Synthetic (derivative of oleanolic acid)	Modification of Keap1 cysteine residues	(i) Efficacy in short-term clinical trials in patients with type II DM and CKD [107]
			(ii) Did not reduce risk of end-stage renal disease (ESRD) or death from cardiovascular failure in patients with DM and stage IV CKD and was terminated early due to side effects [106]

Tert-butylhydroquinone (tBHQ)	Synthetic (preservative in unsaturated vegetable oils and edible animal fats)	Modification of Keap1 cysteine residues/activation of upstream kinases	(i) Prevented glucose-induced impairments in diabetic retinopathy [142]

Cinnamic aldehyde (CA)	Natural (found in bark of cinnamon tree)	Activation of upstream kinases	(i) Lowered blood glucose, total cholesterol, triglycerides, and increased HDL^∗^ in diabetic rats and mice [143]
			(ii) Prevented development of hypertension in conditions of insulin resistance [144]
			(iii) Improved renal and glomerular function [110]

Resveratrol (RES)	Natural (polyphenol, found in the skin of red grapes, peanuts, and berries)	Activation of upstream kinases	(i) Reduced blood glucose and HbA1c^∗∗^ levels [145]
			(ii) Restored secretory function of *β*-cells in response to toxicity [146]
			(iii) Renoprotective effects in DM [147]

Magnesium lithospermate B (MLB)	Natural (active polyphenol acid of *Radix Salviae miltiorrhizae* herb)	Activation of upstream kinases	(i) Suppressed progression of renal injury in diabetic rats [148]
			(ii) Protection against DM-related atherosclerosis [149]

MG132	Synthetic peptide aldehyde	Proteosome inhibitor	(i) Renoprotective against DM-induced oxidative damage, inflammation, and fibrosis [113]

Pterostilbene	Natural (blueberries, grapes)	Mechanism unclear	(i) Protective against *β*-cell apoptosis [150]

Catechins	Natural (flavenols, found in red wine, berries, grapes)	Likely activation of upstream kinases	(i) Reduced hepatic glucose production and enhanced pancreatic function [151, 152]
			(ii) Decreased cytokine-induced *β*-cell damage *in vitro* [153]
			(iii) Prevented reduction in islet mass *in vivo* [154]
			(iv) Protected against nephrotoxicity [155]

^∗^HDL: high-density lipoprotein; ^∗∗^HbA1c: a marker of chronic hyperglycemia.
